# Anæsthetics for Dental Operations

**Published:** 1869-12

**Authors:** Walter Bruce


AMERICAN JOURNAL

OF

DENTAL SCIENCE.

Vol. III. THIRD SERIES--DECEMBER 1869. No. 8

### ARTICLE I.

### *Anæsthetics for Dental Operations.*

### Ether, Chloroform and Nitrous Oxide.

By Walter Bruce, D. D. S.

(*Concluded.*)

The excited condition of the nervous centres, is shown by the talking, laughing, shouting, sometimes even raving, and in nervous individuals, hysterical phenomena; by struggles and attempts to rise, also by the voluntary muscular system in nervous twitchings, tremors, cataleptic symptoms, and rigidity of the muscles, which may extend to the larynx, when death becomes imminent from want of air. These symptoms of excitement vary in different individuals, in some being prolonged and well marked, in others very transient; the patient soon falling into a quiet slumber. They are succeeded by symptoms of insensibility, unconciousness and relaxation. The wild cries become indistinct mutterings, the speech thickens, the pulse sinks, the skin becomes cool and moist, the breathings slow. The pulse, however, rarely falls below its normal standard, even though the agent be administered for a considerable length of time. The mutterings cease, muscles become relaxed, respiration still slower and sometimes stertorous, the reflex function of the spinal cord and the power of perceiving and responding to external impressions by the brain, is entirely suspended and insensibility is complete. This is the stage in which the operation is generally performed, and it may be kept up by from time to time applying the anæsthetic. It is of short duration and vanishes very quickly after the breathing of the vapor has ceased. Upon the admission of air the patient soon recovers, amazed and confused. Sometimes they laugh or weep and the sensibility is generally morbidly keen. Though the use of the senses soon returns, their perfect utility is slowly regained, and there remains for several days a feeling of languor and general *malaise*, and a disagreeable ethereal odor and taste. The stage of insensibility may easily be tested by touching the conjunctiva or edge of the eyelid, if there is no responsive movement of the lid we know that the reflex functions of the nervous centres are suspended and insensibility must be established. It requires about an ounce or two of ether, or a drachm of chloroform to produce anæsthesia. Chloroform usually produces its effect in two or three minutes. Ether is more uncertain, generally, however, requiring about from three to five minutes, not unfrequently ten or fifteen. These agents should not be administered after a full meal or long fast. If the stomach is full it interferes with the movemeuts of the diaphragm which is to the principal muscle or agent of respiration during the anæsthesia, and vomiting is also apt to be produced. The best time is after a full meal has been digested, as the stomach is then empty and the patient is not weak from want of food. The indications which prohibit the administration of general anæsthetic agents are, diseases of the heart, lungs, brain, kidneys, liver, or any disease which has a tendency to exhaust the nervous supply prevent the proper aeration or circulation of the blood or vitiate its condition. If we administer an anæsthetic to a patient with a diseased brain we may expect paralysis of the respiratory muscles; if to a patient with heart disease, syncope; or if with lung disease, asphyxia. Liver and kidney diseases are sometimes attended by dropsy, and may produce death, either by coma, syncope or apnoea, under the influence of the anæsthetic. It would probably be speaking more correctly or exactly to say, instead of apnoea or want of breath, stagnation of the blood in the pulmonic capillaries. Death may take place by apnoea when in the stage of excitement the rigidity of the muscles extends to the larynx closing the rima glottidis and effectually excluding the air from the lungs. We should remember that the blood in the arteries as well as in the veins has been made impure by the inhalation, and that in order to the extrication of the neurine or nerve force, it is necessary that pure oxygenated blood come in contact with the brain substance, and without the extrication of nerve force the heart and lungs cannot be made to act. Syncope is much more to be dreaded than either apnoea or coma. Coma is produced by chloroform or ether in the same manner as it is by opium or any other narcotic poison, namely, by suppressing the functions of the medulla oblongata. If now artificial respiration can be produced and kept up until the narcotic agent is sufficiently eliminated from the system to enable the medulla to perform its office unaided the patient is saved. This has been done in experiments on dogs, and in cases of opium poisoning in human individuals. In apnoea the heart may continue pulsating after respiration has stopped, and resuscitation can be more easily accomplished than in syncope where the heart has ceased to beat and where we have also a benumbed nervous system to stimulate. One of the laws of physiology is that the blood, in order to be kept in the fluid state must be in motion in contact with a living surface. If then motion ceases in the heart, and the blood remains stationary in it long enough for its fibrine to become formed into a clot it is impossible, with the patient in an anæsthetized condition for resuscitation to be accomplished. And the profuse hæmorrhage attendant upon some operations, such as resection of the superior maxillary, increases the coagulability of the blood. If, in the stage of excitement, rigidity extend to the muscles of the larynx, the anæsthetic should be withdrawn and free admission of air allowed, a few inhalations of which will generally cause the patient to become relaxed and fall back in a state of insensibility. If, however, the rigidity is so great as to entirely close the glottis, the tongue should be seized with the fingers, tenaculum or forceps and forcibly drawn forwards, which will have the effect of opening the air passage, and upon the admission of air, relaxation soon succeeds. So also, when more of the anæsthetic vapor has been inhaled than can be absorbed, producing symptoms of apnoea or what is commonly known as asphyxia, livid countenance, abdominal respiration, &c., the vapor is to be withdrawn and air admitted, when the respiration soon becomes natural. In cases of suspended animation resulting from the administration of chloroform or ether, we rely almost exclusively on means adapted to restoring the functions of respiration and circulation. Fresh cold air is to be admitted to the patient, cold water dashed upon the face, stimulating enemata administered, and stimulating embrocations rubbed upon the extremities and surface, on the chest and along the spine. Caustic ammonia applied to the skin, or, what is better a tumbler full of boiling water with a towel stretched over the top, inverted on the patients breast. Shocks from a galvanic battery have been used also, and a great number of other remedies for restoring a patient in such a condition. Our chief reliance, however, is upon means used to produce artificial respiration, compressing the chest with the hands or split sheet and allowing it to expand again, rolling the patient from side to side, raising and lowering the arms having them at the same time brought slightly to the front, in short any means we can devise by which breathing can be simulated. The best means of producing artificial respiration is with the bellows, or with the lungs and mouth of an individual. The nozzle of the bellows is introduced into one nostril, the other and the mouth being closed, and the larynx being pressed back in order to close the esophagus, the lungs are then carefully inflated and the chest afterwards compressed or else the walls of the chest allowed to contract from their own natural elasticity. It is asserted by some that artificial respiration can be still better performed by the operator placing his own mouth over that of the patient, observing the precautions just mentioned, and inflating the patients lungs in that way. M. Ricord has saved several individuals by this method, in whom life was so far gone that the pulse and respiration were suspended. A comparison between ether and chloroform has already been made. Ether is much more frequently used in this country than it is in Europe where chloroform is used almost exclusively. It is here considered a safer anæsthetic than chloroform. A much greater number of deaths from the use of the latter agent are published than from the use of ether. Chloroform, however, is more extensively used and is a more powerful agent than ether. It requires less of the chloroform than of the ether to produce the anæsthetic effect. Insensibility is induced in a shorter time with the former agent and its odor is more agreeable. The anæsthetic and after effect of ether are of shorter duration, and its advocates maintain that it is less dangerous. Chloroform has been administered by Prof. Simpson 15000 times (had been several years ago) without any fatal result. It was administered by Mr. Syme 5000 times and in the Crimean war 25000 times without an accident, except probably one case in this war in which an impure article was used. So also in the Italian war and in the Confederate army, for the first three years (which is as far as I have knowledge) no unfavorable result from the use of chloroform has been reported. It has been computed that one fatal case has occurred in every 16000 in which chloroform has been administered. The computation for ether is still less, and in most if not all the cases in which death has occurred, it was due either to impurity of the article or mal-administration. It is in minor operations such as the dental surgeon has to perform, that the majority of the fatal cases have occurred, operations too in which the anæsthetic is administed more on account of the timidity of the patient than to prevent shock to the system or any great suffering. So that although these agents have been administered so frequently with good results and turned to such good account in general surgery, the dentist does not find in either of them such lan anæsthetic as he would desire, or agents that so nearly approach that desideratum as the next we are about to consider, viz: Nitrous Oxide.

Nitrous Oxide differs from ether and chloroform in being a gas, and in having to be manufactured by the operator, requiring a more or less complicated apparatus for the purpose. Nitrous oxide was discovered in the year 1776 but it was not until 1800 when Sir Humphrey Davy experimented with it, that much of its properties became known. It has been long known under the name of laughing gas, for its peculiar excitant effects when moderately inhaled. When pure it is a colorless inodorous gas with a sweetish taste. It is exceedingly difficult, however, to obtain the gas so pure as to be inodorous. Its symbol is N O, being the protoxide of nitrogen. Its combining proportion 22, and its specific gravity 1569, air being 1000. Nitrous oxide is not a permanent gas but can be reduced to a clear colorless liquid under a pressure of thirty atmospheres at a temperature of 32° Fahrenheit. The liquid differs from some of the other liquified gases in being slowly converted into a gas again; liquified carbonic acid for instance, is converted into a gas with an explosion. The slowness of the conversion of the liquid nitrous oxide into a gas is accounted for in the following way: rapid evaporation takes place from the surface of the liquid, and the latent heat required for the change of state is absorbed from the body of the liquid beneath, thus keeping up in it a sufficient degree of cold to prevent its assuming at once a gaseous condition with an explosion. The liquid then is always intensely cold. The most intense artificial cold yet known has been produced with this liquid. If a drop of it be let fall on the hand it produces a burning sensation owing to the intense cold. It mixes indefinitely with alcohol and ether. If whilst the liquid is evaporating water be dropped upon it the water is frozen and the liquid converted into a gas with an explosion. Exposed under the bell glass of an air pump it becomes a snow-like solid. The curious experiment of freezing water in a red-hot crucible has been done with nitrous oxide liquid, and mercury can with it be reduced to a malleable condition. Sodium and potassium, metals which have a powerful affinity for oxygen are not oxydized in this liquid, but charcoal unites with its oxygen with such rapidity as to produce combustion, nitrous oxide is a supporter of combustion, bodies burning more rapidly in this gas than in the air. If it be mixed with an equal volume of hydrogen in Cavendishe's endiometer and fired with electrical sparks it explodes with violence and liberates its own measure of nitrogen, water being formed by the combination of the hydrogen and oxygen. Water absorbs four times its bulk of nitrous oxide.

Nitrous oxide may be made by the action of nitric acid on zinc in the following way: Thirteen parts of nitric acid are poured on ten parts of zinc. The result is the formation of nitrate of zinc, two parts of deutoxide of nitrogen and one part nitrous oxide. This process may be illustrated by the following formula: $10\ zn + 13\ NO_5 = 10\ (znO\ NO_5) + 2NO_2 + NO$. The deutoxide of nitrogen may be converted into protoxide by allowing it to stand over moist zinc which absorbs one part of the oxygen leaving nitrous oxide. This is a tedious mode and one by which it is rarely made; it is always manufactured for anæsthetic purposes by the fusion of nitrate of ammonia. Nitrate of ammonia is made by pouring nitric acid on carbonate of ammonia.

If nitrate of ammonia be thrown on porcelain heated to about 400° it melts, ebullition takes place and fumes composed principally of nitrous oxide are disengaged. If the porcelain be heated to a red heat the nitrate of ammonia burns. Thrown on a piece of copper heated to a certain degree it is rapidly converted into a gas with an explosion; it is dangerous therefore to undertake to make nitrous oxide in a copper vessel. The fumes disengaged in the fusion of nitrate of ammonia have in them besides nitrous oxide some deutoxide of nitrogen, hyponitric acid, nitric acid, ammonia and probably some others, according to the impurity of the article used. The nitrate is placed in a retort, the retort in a sand bath and heat is applied, the gas which arises is conducted by means of an india rubber tube through purifying solutions into a receiver, from whence it is inhaled by the patient by means of a tube and mouth piece. At the end of the retort there should be a glass bulb attached to receive the water which results from the decomposition of the nitrate of ammonia. Two wash bottles containing, one a solution of sulphate of iron, the other of soda or potassa, have the tubing arranged in them so as to allow the gas to pass through the solutions and be purified. The gas receiver is a large bell-glass-shaped vessel inverted in a cylinder or tube containing water and balanced by means of weights, an apparatus similar to those they have at the gas factories in the cities. The heighth of the inverted vessel in the cylinder indicates the amount of gas we have on band, and the falling of this vessel shows the amount inhaled when the gas is administered. The nitrate of ammonia, from which this gas is made, is composed of 54 parts nitric acid, 17 ammonia and 9 water. Part of the oxygen of the nitric acid unites with its nitrogen and with the nitrogen of the ammonia, forming nitrous oxide, the remainder of the oxygen of the nitric acid uniting with the hydrogen of the ammonia, forming water. The formula for nitrate of ammonia is written $NH_3$ HO $NO_5$ or according to the Berzelean method $NH_4$ O, $NO_5$; upon the application of heat a decomposition takes place and we get a result which may be expressed in the following way 2 NO+4 HO or two parts of nitrous oxide and four of water. There is always some of the deutoxide disengaged in the manufacture of nitrous oxide and if the retort is too rapidly heated, red fumes of this gas may be seen in it. The deutoxide may be got rid of by passing the gas through a solution of sulphate of iron; one part of the oxygen of the deutoxide uniting with the iron forming a sulphate of the sesquioxide. Nitrous oxide is also liable to impurities, (principally chlorine), from an adulterated article of nitrate of ammonia used in its manufacture. The nitric acid with which the nitrate is made being impure from admixture of sulphuric acid, and from the nitrate being sometimes adulterated with muriate of ammonia. When sulphuric acid is present in the nitrate of ammonia it may be detected in the following manner: Make a solution of chloride of barium and pour it into a solution of the suspected nitrate of ammonia. If there is any sulphuric acid present an insoluble sulphate of baryta is formed, causing the clear, mixed solution to become cloudy; somewhat in the same manner the presence of sal ammoniac or muriate of ammonia is detected; mix a solution of the nitrate of ammonia with a solution of nitrate of silver, if any chlorine be present the solutions become milky from the formation of the insoluble chloride of silver. Chlorine is removed from nitrous oxide by passing it through a solution of soda or potassa, another method of purifying is to allow the gas to pass through lime water in the receiver. Nitrous oxide once used should be allowed to escape as useless. It has been attempted to purify them (the exhalations) for second use by passing them through lime water or some solution to deprive them of carbonic acid. This is poor economy and should not be done. Carbonic acid is not the only impurity in the exhalations, and to purify them so as to render them fit for use again, or in other words the manufacture of nitrous oxide from the exhalations, would involve more time, trouble and expense than manufacturing it at once from the nitrate of ammonia, aside from this is the grave objection of the liability to produce contagious diseases by a use of the inhalations. Prof. Vander Weyde, of New York, after manufacturing the gas condenses it into a liquid in a strong iron cylinder, he allows the evaporated gas to pass from this cylinder into the receiver from whence the patient receives it. He claims a number of advantages for the liquid nitrous oxide, among others its portability and its being rendered pure by condensation. The most common operation for which nitrous oxide is administered is the extraction of teeth. The patient is placed in an operating chair, as near a horizontal position as convenient, a cork is placed between the jaws to prevent spasmodic closure of the jaws during the anæsthesia. The mouth piece or inhaler which should be so constructed as to allow unmixed nitrous oxide to enter the lungs and permit free exhalation, should be applied to the mouth, and it should be so arranged as to prevent the entrance of any air. The nostrils are then closed and the patient directed to freely inhale the gas. We should always closely watch the patient during the administration, particular attention being paid to the circulation and respiration. The requisite degree of insensibility may be determined by touching the edge of the eyelid, as before directed, or by the general degree of muscular relaxation by calling upon the patient to perform some voluntary movement, to raise the arm. In case the administration attempt to result fatally, the gas is to be at once withdrawn, fresh air freely admitted stimulants and other means of restoration before mentioned, under ether and chloroform, to be applied should they become necessary. The conditions which contraindicate the administration of nitrous oxide are the same as those given when speaking of ether and chloroform and need not be repeated. Nitrous oxide as a medicinal agent is a diffusable stimulant, and is used (the aqueous solution) in many cases in place of alcohol. By Dr. Zeigler, of Philadelphia, it is very highly extoled and its use recommended in a great variety of diseases. It differs from the other anæsthetics, ether and chloroform in being a supporter of combustion, when introduced into the system by any means it rapidly increases oxidation and consequently the amount of carbonic acid in the blood. It has so far, proved to be far superior to ether or chloroform as an anæsthetic in minor surgery. Its effect is much more evanescent, the stage of insensibility being more rapidly induced and the recovery therefrom much quicker than that of ether or chloroform. The after effects are also much more transient, there being rarely any disagreeable sensations remaining after it has been taken. Anæsthesia is much more agreeably produced with this agent than with any other anæsthetic. Its inhalation is generally attended with very agreeable sensations, by some the sensations are described as being exquisitely pleasurable and it is rare that a patient refuses a second administration of the gas. Its chief recommendation, however, is its safety. Different theories have been advanced in regard to its modus operandi, and are still being discussed. By some it is thought to produce its effect by simply acting as a negative agent, preventing the elemination of carbon from the system, which remaining in the blood produces the anæsthesia. By others it is thought to act by producing a state of super excitation; the vital functions are excited beyond a normal standard, and the cerebral functions in consequence so deranged as to produce anæsthesia. Nitrous oxide is not a harmless agent as some seem to suppose. Death has been produced by its administration for anæsthetic purposes, and though death when it occured was in cases complicated with serious disease of some of the important viscera, yet we should remember that it is an agent that acts with great power on the human system and should always be administered with great care.

